# Tissue Paper-Based Hydrogels for Soil Water Maintenance and Nitrogen Release

**DOI:** 10.3390/gels11080599

**Published:** 2025-08-01

**Authors:** Ana Carla Kuneski, Hima Haridevan, Elena Ninkovic, Ena McLeary, Darren Martin, Gunnar Kirchhof

**Affiliations:** 1 Department of Soils and Natural Resources, State University of Santa Catarina, Lages 88520000, Brazil; anakuneski@gmail.com; 2 School of Agriculture and Food Sustainability, The University of Queensland, Brisbane 4072, Australia; 3 School of Chemical Engineering, The University of Queensland, Brisbane 4072, Australia; h.haridevan@uq.edu.au (H.H.); e.ninkovic@uq.edu.au (E.N.); e.mcleary@uq.edu.au (E.M.); darren.martin@uq.edu.au (D.M.)

**Keywords:** water retention, cellulose-based hydrogel, nitrogen leaching, available water, biodegradable hydrogel, controlled nutrient release

## Abstract

Hydrogels are widely known for their ability to increase soil water retention and for their potential slow nutrient release mechanism. They have been constantly improved to meet the growing demand for sustainability in agriculture. Research focused on the development of biodegradable hydrogels, produced from industrial cellulose waste, are an ecological and efficient alternative soil ameliorant for the improvement of agricultural land. The objective of this study was to evaluate the impacts of two types of hydrogel (processed in a glass reactor versus a twin-screw extruder) on soils with different textures (clay and sandy loam), testing their water retention capacity, nitrogen leaching, and effects on seed germination. The methodology included the evaluation of water retention capacity at different pressures with different hydrogel addition rates in the soil, leaching tests in columns filled with soil and hydrogel layers, and germination tests of sorghum and corn. The results indicated that the addition of hydrogel significantly improved water retention, especially in sandy loam soils. The hydrogels also reduced nitrogen leaching, acting as nitrification inhibitors and limiting the conversion of ammonium to nitrate, with greater effectiveness in clayey soils. In the tested formulations, it was observed that the hydrogel doses applied to the columns favored nitrogen retention in the region close to the roots, directly influencing the initial stages of germination. This behavior highlights the potential of hydrogels as tools for directing nutrients in the soil profile, indicating that adjustments to the C:N ratio, nutrient release rate, and applied doses can optimize their application for different crops.

## 1. Introduction

Soil water retention is critical in the productivity of agriculture and horticulture practices, particularly in regions facing water scarcity and climate variability. The improvement of soil water retention ensures the accessibility of plant-available water, reduces irrigation demand, and supports microbial activity, thereby contributing to more resilient cropping systems and sustainable land management practices.

Hydrogels are three-dimensional networks of hydrophilic polymer chains that can absorb or retain water or solvents—often hundreds of times its weight [1]. These hydrophilic chains are either physically or chemically crosslinked [2]. Superabsorbent polymers (SAPs) are a type of hydrogel that can be produced from synthetic or natural polymers, and its response to environmental conditions can be designed according to the intended purpose [3]. They have diverse applications, including construction, recovery of natural areas, industrial sectors, medicine and agriculture [4].

In agriculture and horticulture, superabsorbent polymers have been extensively used in growing media [5], seed coatings [6] and potential hydro mulch applications. These materials can potentially improve water availability and retention [7,8] soil porosity and aeration due to swelling and shrinkage during the process of absorption and subsequent release of water [9]. This can then lead to increased water use efficiency. They are also used as soil conditioners and nutrient delivery agents to improve soil porosity, promote seed germination, and establishment [10], and enhance crop yields.

In addition to supporting soil water retention, the use of SAPs can also alter the available nutrients for plants [11]. Certain polymers can reduce nitrogen (N) leaching promoted by rain or irrigation [12], and associated with the slow biodegradation of this material, the effect is to provide a source of nutrients for plants and maintain soil fertility [13].

The resultant effect of the SAPs on soil properties depends on several variables including the polymeric composition, the cross-linking process adopted, the size of the gel granules among other characteristics [14]. After being applied to the soil, the SAPs will respond according to the type of soil, depth, pH, its ionic strength, irrigation method, and the application rate [15].

Furthermore, a hydrogel considered ideal is biodegradable and results in water, carbon dioxide and organic matter, improving soil fertility and porosity conditions [16]. Typically, SAPs are composed of petrochemical-based polymers such as polyacrylate or polyacrylamide. These materials are potentially hazardous for ecosystems as they break down into microplastics [17]. Considering the growing concern to preserve the environment, there is increased interest in academia and industry in the development of a biodegradable hydrogels based on natural polymers [6]. Some natural polymers include polysaccharides, chitosan, alginate, and cellulose [18], which can often be found in either agricultural residues or industrial waste.

This study discusses the investigation of one such hydrogel developed from tissue paper offcuts, which is a significant source of cellulose waste. The hydrogel is produced using the proprietary UQ Biogel Technology. However, the efficient agricultural use of this hydrogel requires understanding on their impact on water holding capacity and nutrient release, specifically, how nitrogen is released from hydrogel when the hydrogel manufacturing process includes the use of this. The hypothesis was that water holding capacity and nitrogen release are affected by the structure of the hydrogel, their application rates and on which soil they are applied on. The objectives of this pilot study were to quantify soil-water-nitrogen interactions for two structurally different yet chemically similar hydrogels. Both hydrogels were created from tissue paper in a reactor, but the second hydrogel was mechanically refined using and extruder which possibly increases porosity due to cellulose fibrillation. The hydrogels, labeled Reactor and Extruder, were applied at varying rates to two different soil textures (clay and loam).

## 2. Results and Discussion

### 2.1. Hydrogel and Soil Properties

The ratio of hydrogel additions to the soil and chemical properties was always based of the ‘water content as-is’, i.e., the water content the hydrogel had when it was delivered to the lab or the air-dry water content or the water content at a suction of 1000 Bar (pF 6). There was no significant difference in air dry water content of both hydrogels (*p* = 0.32) and the average was 0.25 g/g (se = 0.01). Commercial hydrogels can be found with different water contents, such as the PR3005A hydrogel manufactured in France with 5 to 7 g/g, and the Tarawat A100 hydrogel produced in Iran with a water content between 3 and 4 g/g [19]. Other hydrogels are commercially available in dry form and can be hydrated before application, and the choice will depend on the purpose for using the hydrogel.

Water content at saturation was very variable. Similarly to swelling clay soils, this may be due to the time it takes for the hydrogel to fully swell and take up water, and insufficient time may have been allowed for this to occur. However, for practical intents and purposes, allowing too long a time for the soil or hydrogel to take up water is not practicable. Certain hydrogels may take 2 h to swell to 60% of their capacity [20]. Other SAPs absorb the maximum amount of water in 60 min [21], in other cases up to 12 h [22], while other authors used 24 h to swell [23,24] as used in this research. In general, this process will depend on the raw material used, surface area and particle size [2].

**Figure 1:**
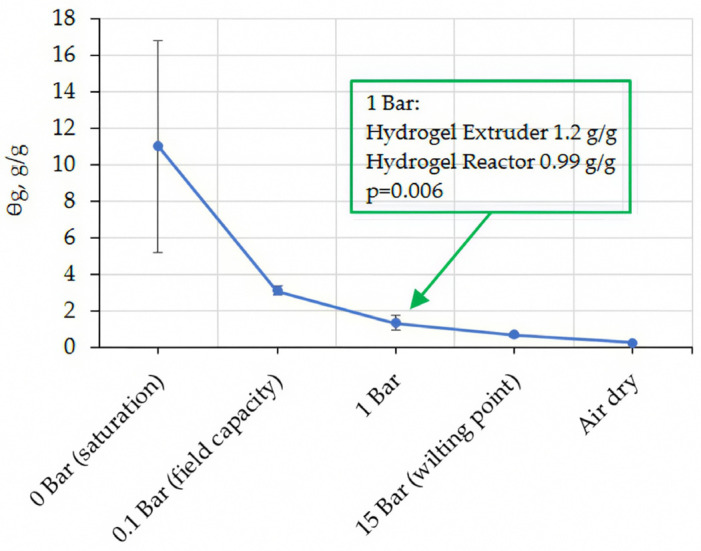
Water release curve of pure hydrogel.

Except for the water content at 1 Bar suction, both hydrogels reactor and extruder had the same soil water release curve (Figure 1). This may be due to slightly more pores in the extruder hydrogel that hold water at that suction. However, this small difference was not considered important in the context of overall water holding capacity.

**Figure 2:**
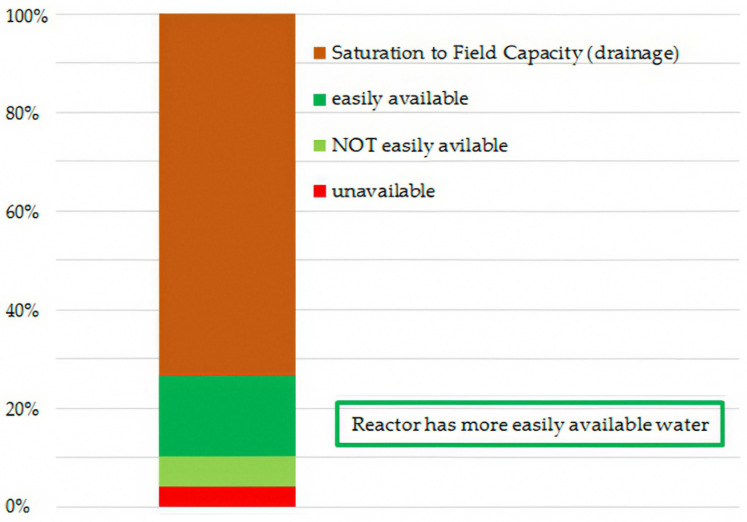
Relative plant available water for the pure hydrogels.

Most of the water found in hydrogels is in the saturated portion and is easily released into the environment when leaving the saturated medium (Figure 2).

There was no significant difference in total carbon (*p* = 0.32) and total nitrogen (*p* = 0.35) content of the two hydrogels reactor and extruder (Table 1). The hydrogels had a very low C:N ratio showing that N is readily available and will not be rendered unavailable during the nitrification process. The C:N ratios of the soils are within the normal range, but the lower C:N ratio of the loam indicates more available N compared to the clay soil.

Hydrogels vary depending on the raw material, chemical properties, preparation method, and the medium with which they interact [25], and therefore, their composition will be variable. Hydrogels with 40.1% carbon and 1.26% have already been reported [26], as well as hydrogels with 22.6% nitrogen [21]. Tests with fertilizers produced for slow release of nutrients and with high water retention capacity produced with ethyl cellulose, show levels of 21.1% nitrogen [27]. Furthermore, the N content is variable in synthetic hydrogels, such as in the form of polyacrylic acid-potassium salts with 0% N and K-acrylate-polyacrylamide copolymer with 14% N [28].

### 2.2. Water Holding Capacity of Soil Hydrogel Mixes

**Figure 3:**
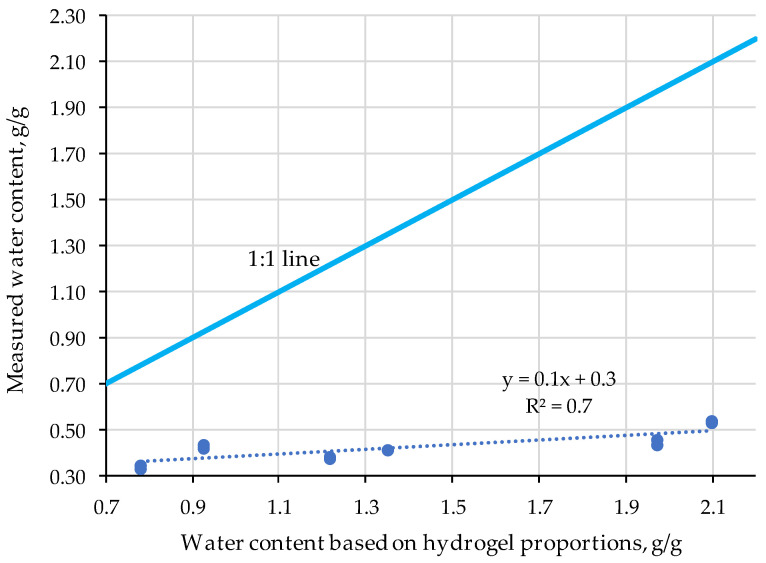
Actual water content of hydrogel soil mixes compared to proportional additions of hydrogels.

Based on the soil water release curve of the soils only and the hydrogels only, we needed to test if the addition of the hydrogels to the soil would alter the water contents at different suction levels proportionally. If this hypothesis was correct, the soil water release curve of the hydrogel-soil mixes can be calculated, and there would be no need to perform extra measurements. Figure 3 proves the null hypothesis. The impact of hydrogel addition is substantially less than would be expected from proportional additions. This observation shows that soil and hydrogel interact and alter the soil structure, and not only hold water as part of a soil fraction. Since hydrogels rely on swelling for water uptake, incorporating them into soil may hinder this ability, and hence reduces swelling, making the water contents at different suction less than what could be expected for a proportional relationship only.

In contrast, when evaluating water retention in clay and sandy loam soils with the addition of 4 and 6 g/kg of the commercial hydrogel Superab A200, the authors obtained as a result an increase in the volumetric water content proportional to the amount of hydrogel applied, with a superior performance in coarse-textured soils with the addition of hydrogel [29]. In our research, applications of 50, 100 and 200 g/kg were evaluated, so the strengthening water content may have been overestimated by the high dose of hydrogel applied. This difference can also be observed in the form of application, which the previous authors applied in dry form, while the hydrogel reactor and extruder hydrogel samples presented 0.25 g/g of water weight.

The application of 0.4, 0.8, 1.2, 2 and 4 g/kg of Stockosorb K 400 hydrogel exponentially increased the water content in the sandy soil, with the effect of retaining high levels of water quantities found in the vicinity of the suction potential on the pressure plates, as well as during the experiment in pots with pine seedlings (*Pinus halepensis*) [30]. Although the retained water does not increase proportionally with the increase in hydrogel application, cellulose-based hydrogels are reported in the literature as effective agents for soil water retention [31,32].

**Figure 4:**
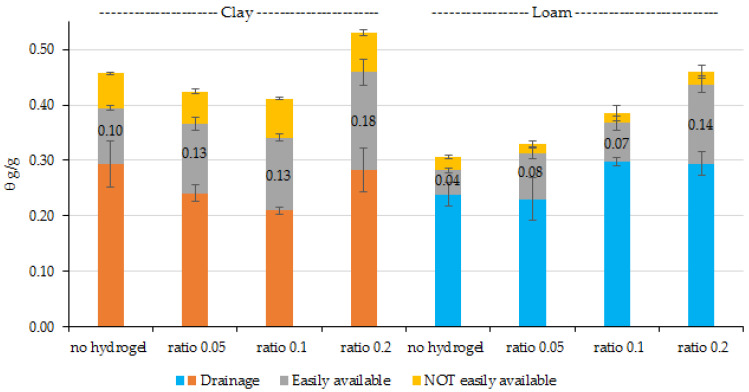
Water holding capacity of the soil: extruder mixes.

**Figure 5:**
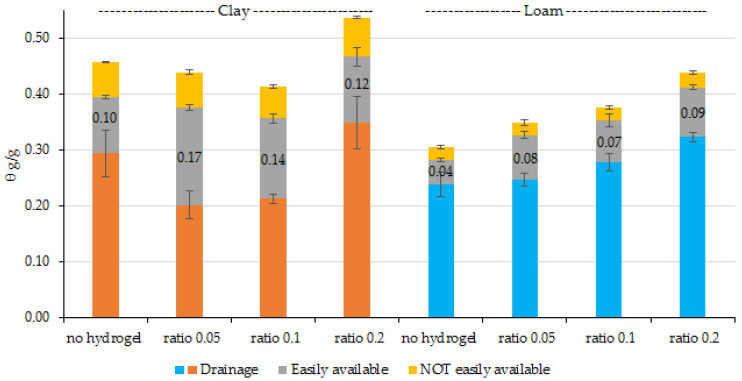
Water holding capacity of the soil: reactor mixes.

The soil water release curves or the hydrogel-soil mixes are given in Figure 4 for the extruder hydrogel and Figure 5 for the reactor hydrogel. The loam showed increasing water contents as more hydrogel was added and little relative change between the hydrogel types. A soil hydrogel ratio of 1:20 doubled readily available water and, within experimental variability, increased further as the ratio increased to 5, with a three-fold increase for the extruded sample, but less for the reactor sample and high hydrogel additions.

For the clay soil addition of hydrogel also increased readily available water. This is attributed to a reduction in drainage and suggested that the hydrogel reduced drainage for the benefit of readily available water, possibly as a consequence of reduced swelling, but only until the hydrogel–soil ratio reached 5.

Soil particle size is an important factor in water retention and varies according to the mineral composition of the soil [33]. In this sense, the dry density of the fine fraction (pd-f) also plays a determining role in the shape of the soil water retention curve (SWRC), especially at lower suctions [34]. According to the authors, SWRC is sensitive to variations in pd-f when the matric suction is below 715 kPa, and this behavior is associated with the predominance of interaggregate pores, which are more susceptible to compaction. At higher suctions, intra-aggregate pores begin to dominate water storage, and SWRC becomes virtually independent of the density of fines. The authors also observed that increasing the dry density of fines reduces the proportion of interaggregate pores, leading to lower water retention capacity at low suctions. In the case of Loam soil, total water retention is lower than in Clay soil, due to the size of soil particles, with less clay and micropores, as well as having less resistance to hydrogel swelling, with the positive effect of increasing the content of easily available water. In this sense, the pronounced and increasing effect of the addition of cellulose-based hydrogels in soils with a coarser texture has already been presented in the literature [35,36,37], by altering the physical structure of the soil by reducing macropores or drainage pores [20].

As for Clay soil, the interaction between soil and hydrogel may have generated a physical change in the soil, with a reduction in water retention due to the blockage of pores by the swelling of hydrogel particles when they expand when absorbing water. This effect has already been reported in the literature, when it was observed that the hydrogel changed the pore distribution and its size to smaller pores [38]. In addition, clayey soils are characterized by greater water retention due to the composition and arrangement of a high proportion of layered silicate particles in the soil with a predominance of small pores [39].

Considering the dose adjustment, the 0.2 ratio of reactor and extruder hydrogel samples could be avoided in clay soil, as their addition mainly increases the water lost through drainage. While in Loam soil, both hydrogels at the 0.2 ratio increased the available water, because due to the granulometric nature of this soil, the hydrogel has a greater capacity to expand and store water.

### 2.3. Nitrogen Leaching

The leaching columns were wetted and after each leaching event were left to drain.

**Figure 6:**
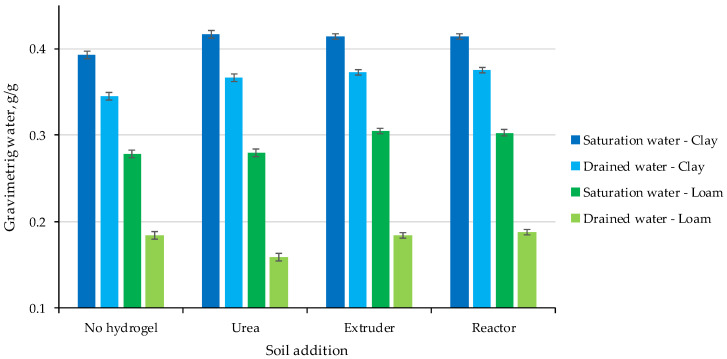
Saturated and drained water contents of the leaching columns.

Although differences in water content were significant between saturated water (*p* = 0.05) and drained water content (*p* < 0.001) for both soils, the main effect was the type of soil added to the column, with loam soil having lower water contents than clay soil and a large difference between saturated and drained water contents (Figure 6).

It is important to note that having a layer of soil mixed with hydrogel or Urea did not have a negative effect on drainage.

This effect can be attributed to the lighter texture and larger pores associated with the sandy soil matrix, while soils with higher clay content have greater water retention capacity associated with greater adsorption and capillary forces [40]. It is important to note that having a soil layer mixed with hydrogel or urea did not have a negative effect on drainage, i.e., the mid-layer did not act as an interruption for water movement. Furthermore, there was an increase in saturation water for both soils with the additives. Similar results were observed with the application of 4 and 6 g/kg of hydrogel in sandy loam and clay soil, in which an increase in saturation water content was observed, as well as an increase in available water content [29]. Significant impact of the use of hydrogel in loamy sand soil was also observed in the pF range 0–2.2, in addition to the increase in total soil porosity and water retention capacity [41].

**Figure 7:**
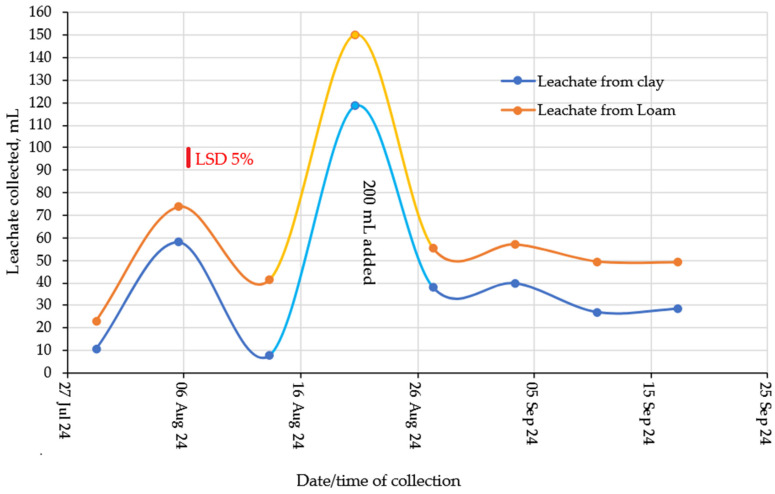
Leachate collected at different times.

The amount of leachate collected at the different leaching events was not affected by the hydrogel or urea treatments but was higher for the loam compared to the clay (Figure 7). Therefore, soil texture was the main factor influencing drainage dynamics, with higher leachate volumes in loam soil in all events due to its greater macroporosity and hydraulic conductivity, which favor water percolation. On the other hand, the greater water retention in clay soil can be explained by the predominance of micropores, which favor retention by matric forces. Furthermore, after the addition of 200 mL of water, there was a reduction in collected volumes, suggesting internal moisture redistribution or greater water retention in the lower soil layers, especially in clay. The reduced leachate collected from the clay was probably due to higher evaporation rates or very slow final leaching rates.

The absence of difference between the addition of hydrogel, urea, and the control may be associated with the low amount of these additives applied in each column, or even due to the start of the test at the saturation point. The mild temperature of the laboratory environment may also have influenced the maximum absorption efficiency of the hydrogel, since high temperatures favor the diffusion of water and the swelling of the particles [25,42].

**Figure 8:**
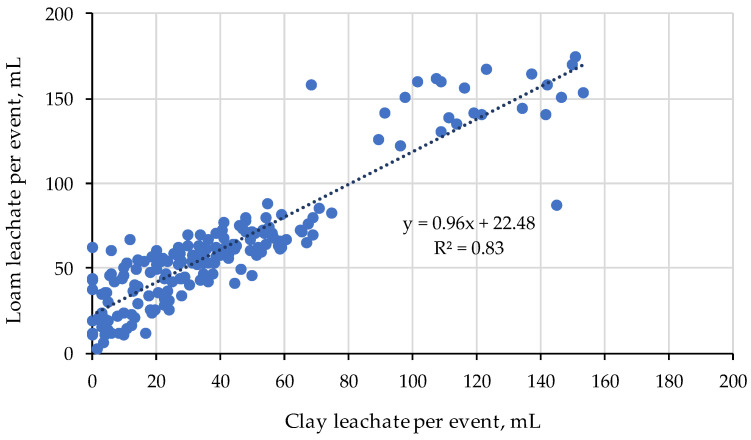
The relation between leachate collected from the clay compared to the loam.

There was a close relationship between the leachate collected from the loam and clay (Figure 8). The intercept of the regression (22 mm, Figure 8) is the average difference between leachate from the loam and clay soils. It suggested that leaching from the clay continues after we assumed that leaching had reached an end point. This may be due to lower leaching rates in the clay due to its lower hydraulic conductivities at high water contents compared to lighter textured soils [43].

**Figure 9:**
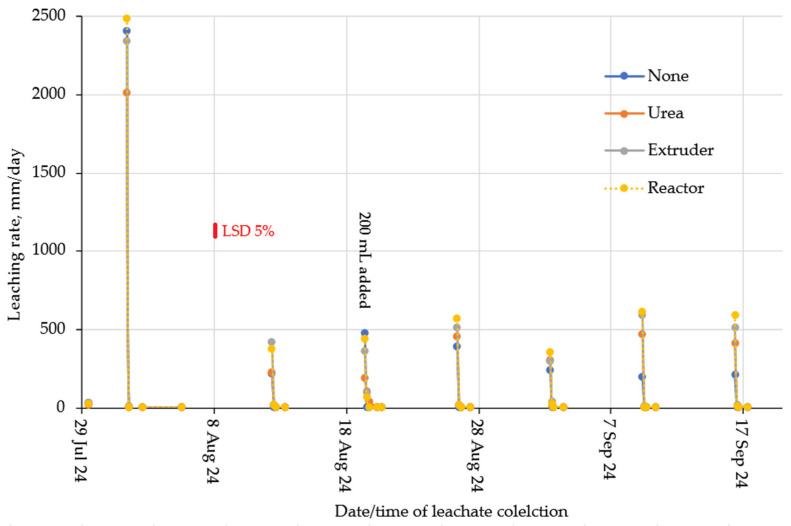
Leaching rates of the loam soil.

**Figure 10:**
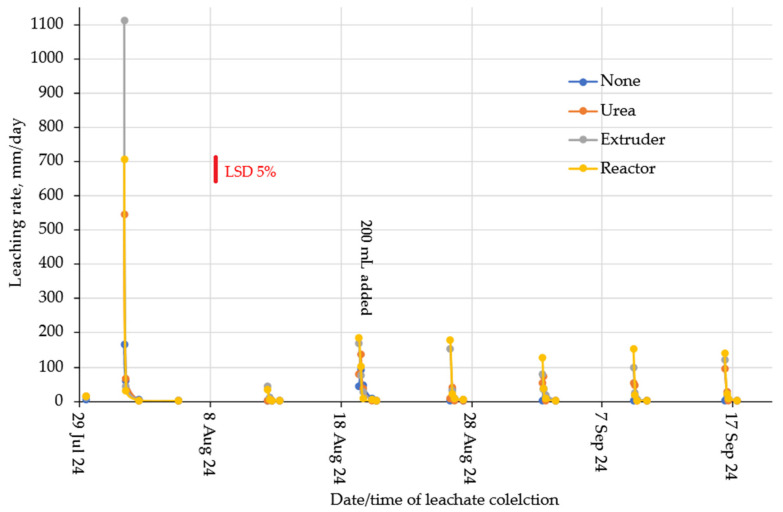
Leaching rates for the clay soil.

Leaching rates for the two soil types are given in Figure 9 (loam) and Figure 10 (clay). Leaching rates were largest at the first leaching event and reduced for subsequent events as the soil slowly consolidated and hydraulic conductivity decreased. For the clay soil, the layer with the reactor hydrogel tended to have higher leaving rates, which was probably due to its more (observed) brittle rather than soft gel-type structure. The same was observed for the loamy soil, except for the first leaching event.

The first leaching event is the water obtained after the columns are removed from the saturated medium. From this point on, the soil particles are organized in the space of the columns and with the leaching events, the particles are redistributed, reducing porosity and water flow [44]. In addition, water evaporates from the soil, which contributes to the reduction in leachate.

**Figure 11:**
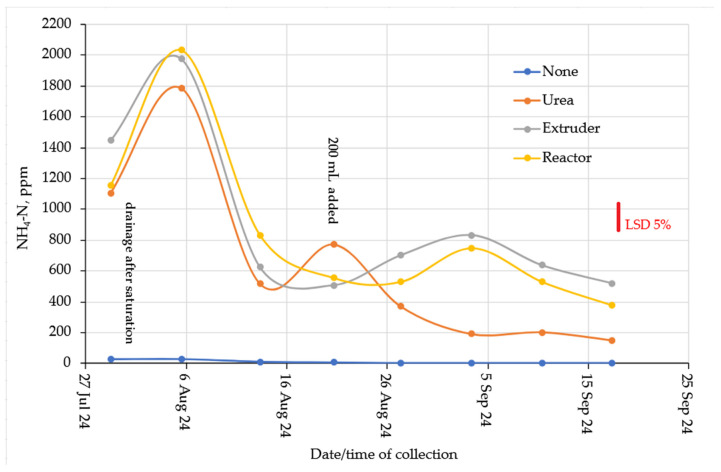
Ammonium-N in the loam columns.

**Figure 12:**
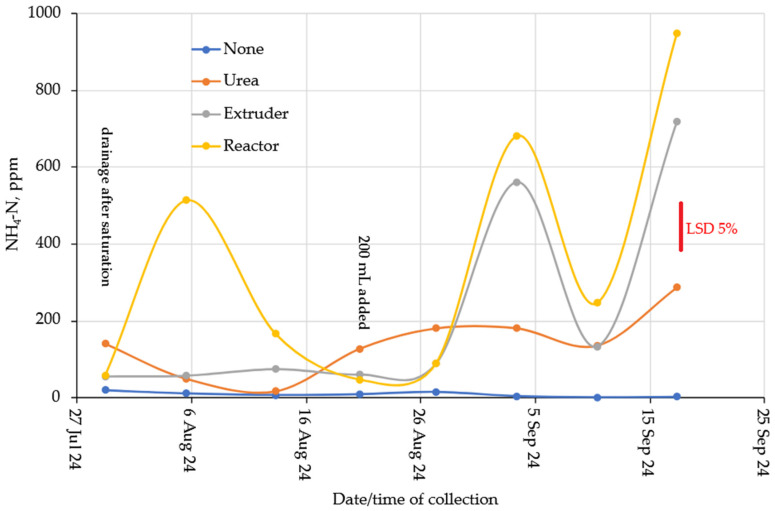
Ammonium-N in the clay columns.

The NH_4_-N concentrations in the leachate from the clay and loam are presented in Figure 11 (loam) and Figure 12 (clay). The leachate from the soils without a layer of urea or hydrogel contained very small amounts of NH_4_-N. There was no significant effect of N-rate (*p* = 0.992), hence no data is presented. On the loam soil, the addition of N from Urea or hydrogel was similar. There was a maximum in the first flush after the column drained, and then concentrations of NH_4_-N declined during subsequent leaching events, but the hydrogels maintained the highest concentrations of NH_4_-N until the end of the leaching experiment, with approximately twice the concentration of NH_4_-N compared to Urea. The release of NH_4_-N from the clay column was much more variable, but concentrations were also significantly lower compared to the loam soil, and the hydrogels had overall higher leaching rates than the loam. The large variability and flushes of NH_4_-N release cannot be explained at this stage.

Hydrogel is considered a tool to improve the efficiency of fertilizer use in soil, through the transport, storage, and release of nutrients [45,46], in addition to reducing leaching losses [47]. The nitrogen released from common fertilizers was 80 to 90% in 2 to 5 days, while the hydrogel released 75% of nitrogen in 30 days [21], corroborating the understanding that the biopolymer has the capacity for slow and gradual release of nutrients through the dynamic exchange between hydrogel and soil solution. In another case, the release of 90 to 99% of urea encapsulated in hydrogel was released 20 to 30 days after application to the soil [48]. Compared to commercial NPK fertilizers, the application of cellulose-based hydrogels shows a slower and more gradual release of NH_4_^+^ [42]. The release of ammonium N, and other nutrients, depends on the crosslinking density and hydrophilic groups of the hydrogel produced and can be adjusted for the proposed use [49].

**Figure 13:**
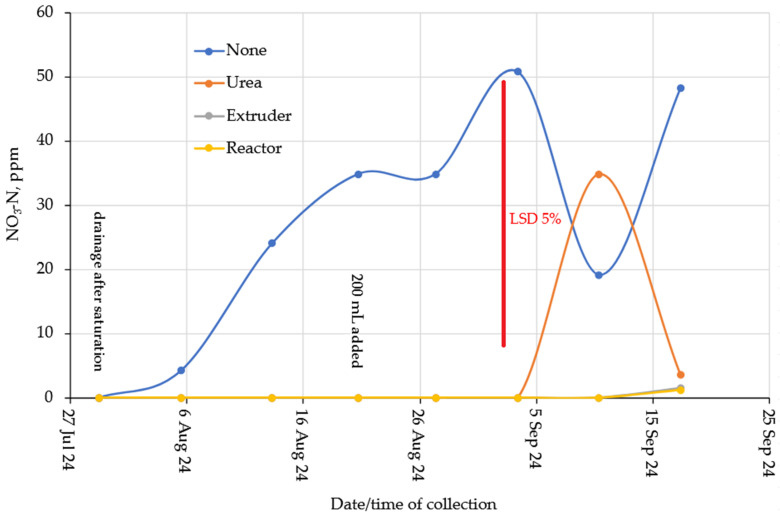
Nitrate-N in the Loam columns.

**Figure 14:**
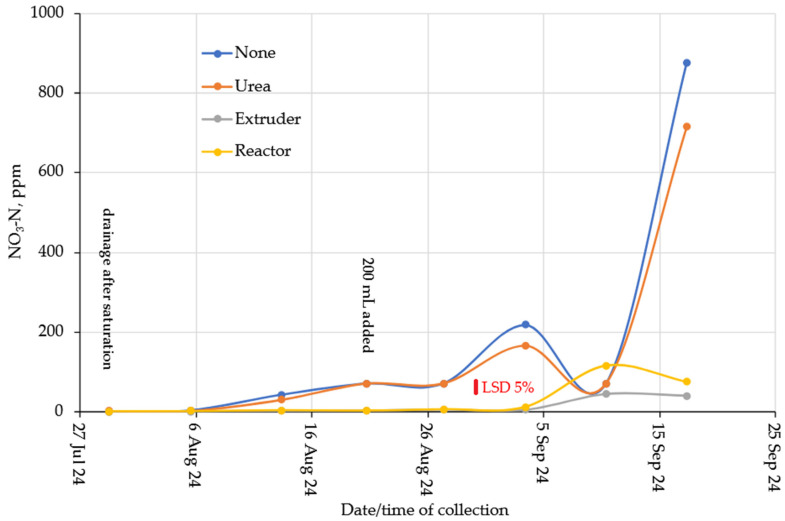
Nitrate-N in the clay columns.

Nitrate concentrations were substantially lower compared to Ammonium (Figure 13 for Loam soil and Figure 14 for Clay soil). However, reasonable nitrification took place in the soil without N-additions. This is probably due to the favorable C:N ratio (Table 1) and a nitrification flush occurred in the Urea columns after about 6 weeks. The complete lack of Nitrate from the hydrogels suggested that they act as a nitrification inhibitor. This conclusion was supported by the observation from the clay columns (Figure 12). From an environmental point of view, biopolymer hydrogels act safely and beneficially in the soil, as they are biodegradable and have a slow-release fertilizer effect [33].

Clay soil without hydrogel or urea addition showed lower release of Nitrate-N compared to Loam soil, which is associated with the greater water and nutrient retention capacity of clays [50]. While urea fertilizer releases 95% of urea in 1 week in moist clayey soil and sandy soil, the addition of hydrogel reduced urea release by 60% after 7 days, indicating that cellulose hydrogels could be used as controlled-release fertilizers in different soils [32]. The same result was observed in sandy soil with the addition of hydrogel, where 72.8% of nitrogen was released in 30 days, while 98.5% of nitrogen was released from urea 12 h after application to the soil [27].

The nitrification inhibitory effect observed with the addition of hydrogels may be associated with the slow release of NH_4_^+^ and a reduction in the activity of nitrifying bacteria due to the physicochemical conditions created. In contrast, the nitrification discharge presented by urea may have occurred when nitrifying microorganisms found better aeration and drainage conditions that favored the conversion of NH_4_^+^ to NO_3_^−^. Urea has low efficiency in nitrogen supply due to its high decomposition and volatilization rate; thus, the hydrogel appears to be an alternative for the slow release of nitrogen [35].

Our results on the use of hydrogels in soil, regarding increased water retention and gradual nitrogen release, can be compared with findings on the impact of adding micro-plastics (MPs) to soil. The introduction of MPs increased soil pH, increasing porosity and aeration, with a consequent increase in biological activity and hydrogen ion consumption [51]. These changes indicate that polymers applied to soil, whether in the form of hydrogels or microplastics, can significantly modify soil physicochemical and biological properties. Furthermore, increased biological activity influences the nitrogen cycle through changes in the abundance of nitrogen-fixing bacteria and the dynamics of antibiotic resistance (ARGs) [51]. Similarly, the application of biochar and polylactic acid to cadmium-contaminated soils increased pH, organic matter content, and total nitrogen [52], due to the release of organic acids and the decomposition of polylactic acid in the soil, which altered the ionic balance and increased the formation of complexes with nutrients and metals. Although the materials used differ, the observed effects reinforce that the use of polymers in soil, such as hydrogels or polylactic acid, can result in changes in soil proper-ties, especially moisture retention, nutrient availability, and pH dynamics.

**Figure 15:**
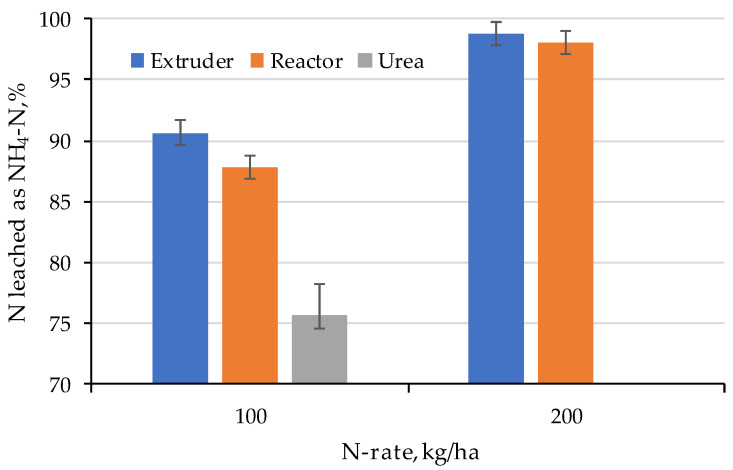
Fraction N leached as Ammonium (note: there was no 200 kg/ha N treatment of Urea).

**Figure 16:**
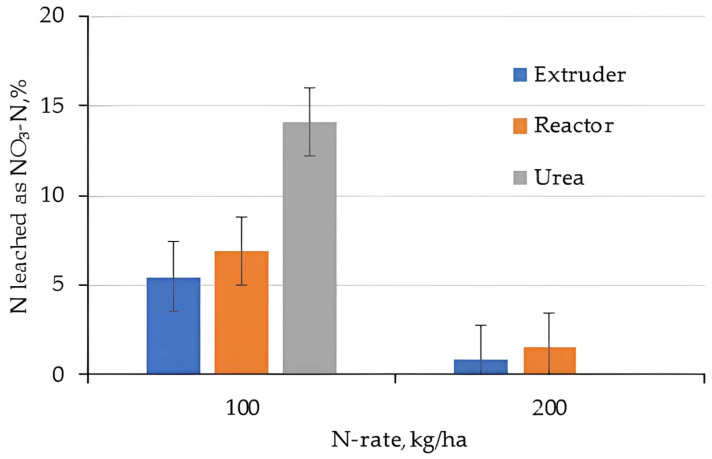
Fraction N leached as Nitrate (note: there was no 200 kg/ha N treatment of Urea).

**Figure 17:**
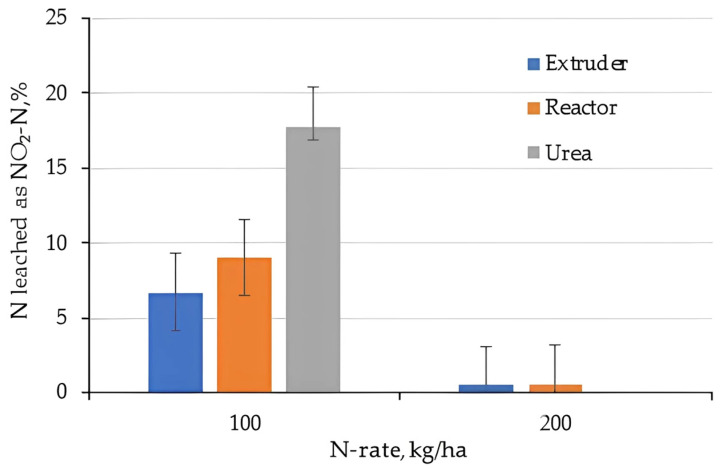
Fraction N leached as Nitrite (note: there was no 200 kg/ha N treatment of Urea).

The total Nitrogen and fractions are given in Figure 15 (NH_4_-N), Figure 16 (NO_3_-N), and Figure 17 (NO_2_-N). Almost all N was leached as Ammonium and the proportion of NH_4_-N as part of the total was highest for the hydrogels. However, doubling the application rate of hydrogels increased the fraction of Ammonium, simply because there was more of it. This can be explained by the fact that hydrogels increase water availability in the soil and facilitate ammonium mobility, with a rapid release of nutrients present on the hydrogel surface soon after swelling, which then tends to be reduced and controlled with the release from the hydrogel matrix [53]. Superabsorbent and fertilizer hydrogels have a nutrient dissolution rate that depends on the physical properties of the material, such as porosity and hydrophobicity; the more porous and hydrophilic the material, the greater the release of NH_4_^+^ [27].

Nitrate as a proportion of total N leached was higher for the 100 kg/ha N treatment compared to the 200 kg/ha treatment, and was higher for Urea compared to hydrogel. This further supported the hypothesis that hydrogels act as nitrification inhibitors, in particular if applied at high rates. Granular urea is characterized by its high N concentration and rapid solubilization and, consequently, has low efficiency due to losses by leaching and volatilization [54]. To overcome this scenario, the use of hydrogel-coated urea was applied at a rate of 2% in sandy soil; the results indicate its efficiency in reducing N losses by limiting the nitrification rate and losses by nitrate leaching [55].

Nitrite is the intermittent product of nitrification when NH_4_^+^ oxidizes to NO_3_^−^ via NO_2_^−^. Very little Nitrite was observed in the high N-application rates from hydrogels and the differences in Nitrate at low N-rates for Urea and hydrogel applications.

**Figure 18:**
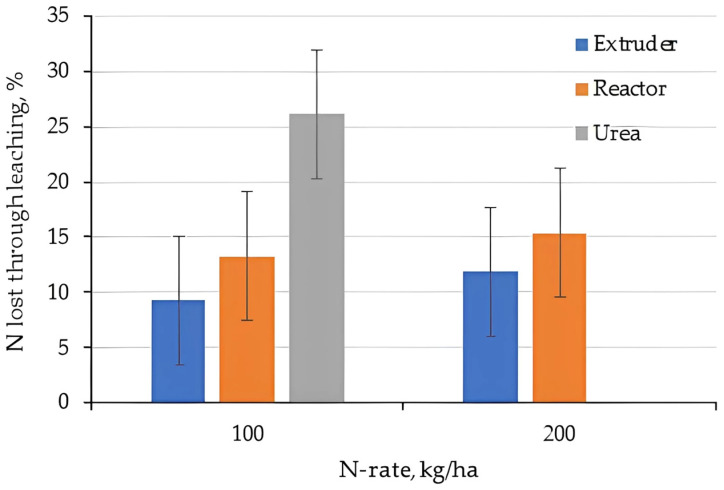
Total N lost compared to N-applied for the clay.

**Figure 19:**
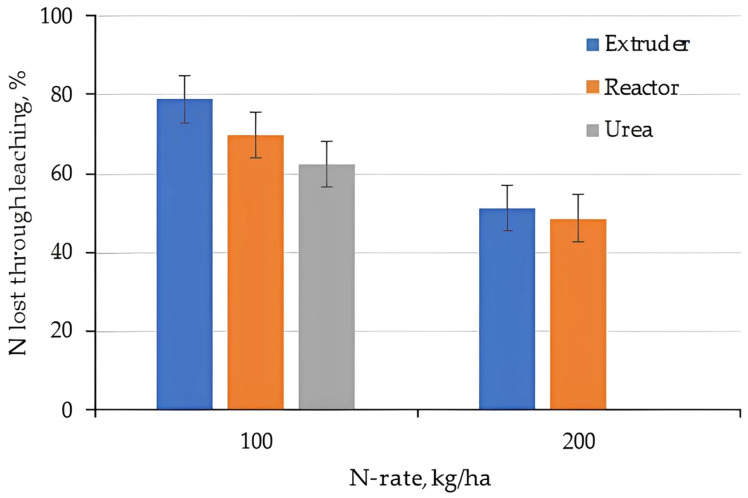
Total N lost compared to N-applied for the loam.

The total amount of N lost during the leaching experiment is given in Figure 18 (Clay) Figure 19 (Loam). Applying N as Urea on the loam resulted in ~25% loss after 8 leaching events in 2 months. This was due to leaching of Ammonia as well as Nitrate as the Urea nitrified to Nitrate. In the presence of water, pure urea is dissolved in a few seconds, which justifies the rapid leaching of N forms [56].

Total N loss was smaller if N was applied as a hydrogel, but still between 10 and 15%. The relative difference between the low (100 kg/ha) and high N rate (200 kg/ha) was small, but given the higher absolute rate, Ammonium leaching increases with hydrogel application rates. Hydrogels produced based on the biopolymer chitosan and casein to carry urea to the soil released 80% of the urea after 50 days applied to the soil, reinforcing the gradual release effect of nutrients [57].

Overall, N-loss in the loam was much higher compared to the clay which is due to the higher hydraulic conductivities and less retention on the low cation exchange complex of the sandy soils [58].

### 2.4. Seedling Growth

**Figure 20:**
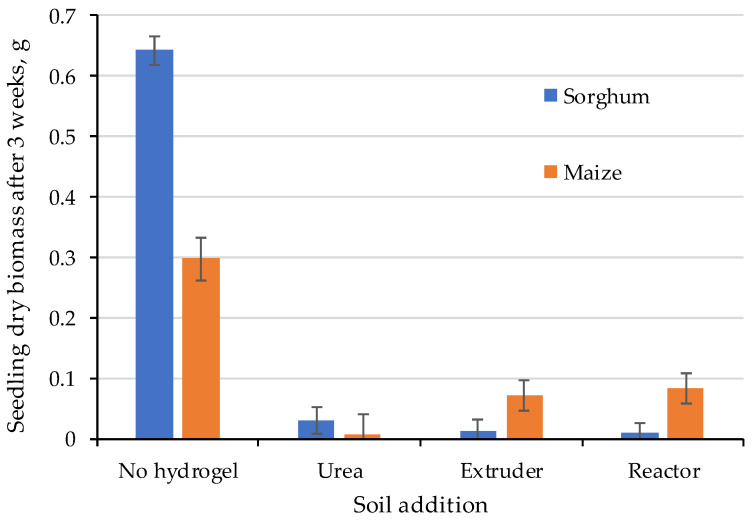
Seedling growth after 3 weeks as affected to N-addition from different sources.

The growth of sorghum and maize seedlings (Figure 20) was strongly affected by the application of any type of N-sources. Even a band of Urea had a detrimental impact on seedling growth, even more so than the hydrogels. This could result from the radical reaching the nitrogen band before the first leaves emerge. For maize it takes about 7 to 12 days for the radical to grow to 6–12 cm depths, and for sorghum it takes 5 to 10 days for the radical to grow to 4 to 10 cm depth. We ran the seedling test for 3 weeks and it is most likely that the radical entered the N-band and the associated high N-concentration killed or severely affected the seedling growth. Under field conditions this is less likely to occur as the fertilizer band is not directly underneath the seed and seeds may also bypass the high band of fertilizer.

The effect of the commercial hydrogel cross-linked potassium poly-acrylic acid on the physical properties of the soil and on the growth rates of corn in pots, using sandy and silty clay loam soils was evaluated [8]. The authors observed an increase in plant height as the hydrogel concentrations increased for the different soils tested. The hydrogel was responsible for reducing water losses through evaporation, increasing water retention in the soil, as well as corn growth. The favorable effect of the hydrogel added to the soil on water retention and plant development has already been reported by several authors [30,59,60,61].

In contrast, in a continuous experiment in a nursery, the effect of different fertilizations with Aleppo pine seedlings was evaluated [62], the authors found a negative relationship between seedling survival and hydrogel application, which may be associated with seedling quality, insufficient dose, and higher soil salinity generated by the release of nutrients from the hydrogel, since the water content is similar to the control soil, but with the negative effect of seedling mortality. Our results show the negative effect of the hydrogel on seedling germination and establishment, probably due to increased soil acidity, resulting from the high content of ammonium, nitrate and nitrite released by the hydrogel and urea [63].

## 3. Conclusions

This study highlights the potential of biodegradable hydrogels produced from industrial cellulose waste as sustainable soil amendments to increase water retention and minimize nitrogen loss. The incorporation of hydrogels increased the water retention capacity of clay and loam soils, with a pronounced effect in loam soils. In clay soils, the addition of hydrogel reduced nitrogen leaching, likely due to its interaction with the denser matrix which reduces water movement in the soil profile. The use of high doses of hydrogel near the root zone can release excess nitrogen, requiring optimization of the hydrogel’s C:N ratio.

Future research challenges include how practical swelling times and impacts on seedling growth require optimization for field applications. These findings support the development of hydrogel technologies for sustainable agricultural practices. Although hydrogels improve water retention capacity and act as nitrification inhibitors, their effects depend on the soil and are influenced by application rates.

## 4. Materials and Methods

### 4.1. Soils

This study utilized sandy loam topsoil from a duplex profile at the Gatton Nature Reserve and a loamy clay from the crop research unit at the UQ Gatton campus. We will refer to the lighter textures soils as a ‘Loam’ and the heavier texture soil as a ‘Clay’. Soil samples were air dried following collection and sieved to pass a 2 mm mesh.

### 4.2. Hydrogels

The hydrogels were produced using the proprietary UQ Biogel technology from tissue paper offcuts (bleached cellulose feedstock), received from Queensland Tissue Products (Queensland Tissue Products, Brisbane, Australia). The hydrogels were made in a reactor vessel under confidential conditions, and one of them was further refined using a twin-screw extruder (HAAKE PolyLab OS Rheomex PTW 16/40 Thermo Scientific, Birmingham, UK) co-rotating intermeshing twin-screw extruder with a screw diameter of 16 mm and a barrel length to diameter ratio (L/D) of 40:1).

### 4.3. Chemical Analysis of the Soil and Hydrogels

Total Carbon on Nitrogen content of the two soils and hydrogels was measured using dry combustion using a LECO 928 analyzer (LECO Corporation, St. Joseph, MI, USA). Results were expressed on a mass basis.

### 4.4. Soil Water Retention

A pressure membrane apparatus was used to measure the soil water release curves at saturation, 0.1 bar (field capacity or pF 2), 1 bar (pF 3) and 15 bar (wilting point or pF 4.2). Hydrogel was added to the soil at rates that would practically be possible for field application. We used soil to hydrogel ratio of 5, 10 and 20, or expressed as hydrogel to soil ratios 0.2, 0.1 and 0.05. Approximately 40 g of each sample were weighed and placed in a 5 cm diameter ring and compressed at 1 Bar uni-axial pressure to achieve bulk density similar to field conditions. Following with saturation over a 24 h period, the samples were placed on ceramic plate and water extracted at the three different pressures.

After soil samples were equilibrated and were dried at 105 °C to determine the gravimetric water content. The soil water release function was also determined on hydrogel only to assess if the change in hydrogel addition is proportional to the increase in waster holing capacity.

The statistical design was 5 factors: (1) soil at two levels (clay and loams), (2) hydrogel at three levels (None, Extruder and Reactor), (3) application rate at 4 level (0, 0.05, 0.1 and 0.2 part of hydrogel in the soil), (5) water holding capacity at 4 levels (0, 0.1, 1 and 15 Bar). A total of 4 replicates were used. Due to the large number of possible treatment combinations only the most important combinations were used to cover the range of application rates and hydrogel types leading to an unbalanced factorial design.

### 4.5. Leaching Columns

PVC tubes (20 cm length, 7.5 cm diameter) were used as leaching columns. Fertilizer rates of 100 and 200 kg/ha N were used based on rates commonly used rates in broad acre cropping systems. Banding of fertilizer is becoming more common and we based our application rates assuming the column is the fertilizer band of 5 cm width. With a row spacing of 60 cm, there are 167 band in 100 m width and 100 m long, i.e., 0.05 m × 100 m long × 167 band or 833 m^2^. A total of 100 kg per 833 m^2^ therefore equates 0.53 g of N over the area of the leaching columns to obtain an equivalent rate of 100 kg/ha banded. The application rates we used were based on this N concentration.

Nitrogen was added in the form of hydrogel. A Urea treatment was included to compare hydrogel with standard fertilizing methods and a control without N added. We used a hydrogel soil ratio of 10 as the most practical application rates. At first around 10 cm of soil was added and slightly compacted by gently tapping the columns to consolidate the soil. Then the Nitrogen soil mix was added to supply the equivalent rates of 100 and 200 kg/ha of N. The columns were then filled with soil. Hence the columns had about 10 cm of soil in the bases and 10 cm of soil in the top with the N in the middle. The base was covered with 100-micrometer nylon screens to stop soil falling out of the columns and allowing water to drain from the columns.

The columns were saturated by immersing them in a water bath. The first leachate was collected following saturation. Then 100 mL of deionized water was added very ~7 days and leachate collected. This gave a total of 8 leaching events. Too little leachate was collected after the 2nd event, and we therefore increased the water application of 200 mL for the 3rd event but then reduced it back to 100 mL. Over the diameter of the columns, these rates corresponded to rainfall events of 23 and 45 mm. The evaporation rates from the cores were around 5 mm per day or 16 mL of the 100 mL of water added. Rates of leaching were monitored until leaching approached zero.

The leachates were analyzed for NO_3_-N and NH_4_-N using MQuant test strips ((Merck KGaA, Darmstadt, Germany)). If concentrations were above range, the leachate was diluted. The results from the test strips were compared and calibrated against the colorimetric methods for NO_3_-N and NH_4_-N used by the Centre for Water and Environmental Biotechnology at the University of Queensland. Due to financial constraints only half of the leachates were used for this comparison. The nutrients ammonia (sum of ammonia and ammonium), nitrite and NOx (sum of nitrate and nitrite) were analyzed on a Lachat QuikChem8500 Flow Injection Analyzer (Lachat Instruments, Hach Company, Loveland, CO, USA) [64,65].

After 8 leaching events, sorghum seeds and then maize seeds were sown to evaluate the effect of hydrogel on seedling establishment. Germinations rates for sorghum were 95%, for maize 85%. Two seeds were sown and then thinned to one after emergence.

The leaching assessment was a randomized design with 4 factors: (1) soil at two levels (clay and loams), (2) hydrogel types at four levels (None, Urea, Extruder and Reactor), (3) N application rate at 3 levels (0, 100 and 200 kg/ha). A total of 4 replicates were used. Due to the large number of possible treatment combinations only the most important combinations were used leading to an unbalanced design. A total of 8 leaching events approximately 1 week apart were monitored. A repeated measures ANOVA was used to analyze the different leaching events.

GenStat Twenty-fourth Edition with the GenStat Procedure Library Release PL32 software was used for all statistical analysis.

## Figures and Tables

**Table 1:** Total Carbon and Nitrogen content of the hydrogel (reactor and extruder) and soils.

Material	%C (se)	%N (se)	C:N Ratio
Hydrogel	31.1 (1.6)	19.6 (0.6)	1.6
Loam	1.15 (0.09)	0.09 (0.01)	13
Clay	1.67 (0.09)	0.07 (0.01)	24

## Data Availability

All data generated in this study is held by the research team.
